# Integrative Drug‐Target Causal Analysis, Single‐Cell Sequencing and In‐Vivo Validation for Dissecting Molecular Mechanisms Underlying Focal Epilepsy

**DOI:** 10.1002/cns.70985

**Published:** 2026-06-15

**Authors:** Huaiyu Sun, Xuewei Li, Weixuan Zhao, Hongmei Meng, Wuqiong Zhang

**Affiliations:** ^1^ Department of Neurology The First Hospital of Jilin University Changchun Jilin China; ^2^ Department of Radiology The First Hospital of Jilin University Changchun Jilin China

**Keywords:** drug interactions, focal epilepsy, genetic association, mendelian randomization, Quantitative trait loci

## Abstract

**Aims:**

To identify and verify new drug targets for focal epilepsy.

**Methods:**

We combined single‐cell expression data from GSE190452, with genetic data from the eQTLGen alliance and utilized expression‐associated single nucleotide polymorphism as an instrumental variable in Mendelian randomization analysis to investigate the causal link between gene expression and focal epilepsy risk. Moreover, co‐localization analysis was used to evaluate the genetic mediating effect of gene expression. Potential drug interaction mechanisms involving the protein products of key genes were explored using molecular docking technology. The results were verified using an animal model of temporal lobe epilepsy.

**Results:**

The results of Mendelian randomization analysis found that four genes (CASP1, FST, IL10RA, and SUCNR1) were significantly associated with focal epilepsy risk in the FinnGen and UK Biobank cohorts. However, only SUCNR1 (odds ratio [OR] = 0.462; 95% confidence interval [95% CI]: 0.240–0.890; *p* = 0.021) and IL10RA (OR = 0.719; 95% CI: 0.547–0.945; *p* = 0.018) showed consistent negative correlation, indicating that they may have a protective effect (OR < 1). Meanwhile, CASP1 (OR = 1.260; 95% CI: 1.023–1.553; *p* = 0.030) and FST (OR = 1.377; 95% CI: 1.044–1.816; *p* = 0.024) were associated with increased risk (OR > 1).

**Conclusion:**

CASP1, FST, IL10RA, and SUCNR1 are potential druggable genes and promising therapeutic targets for focal epilepsy treatment.

## Graphical abstract

Through Mendelian randomization analysis of the quantitative trait locus data, we identified that four drug targets, namely CASP1, FST, IL10RA, and SUCNR1, have significant genetic associations with the risk of focal epilepsy. This analysis provides preliminary genetic evidence for the causal mechanism by which these targets are involved in the onset of epilepsy. This discovery suggests that intervention strategies targeting the above‐mentioned targets may offer new potential directions for the precise treatment of focal epilepsy. This was created by Figdraw.

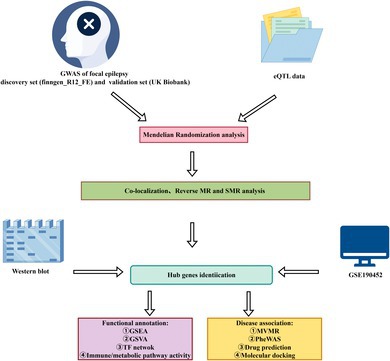

## 1 Introduction

World Health Organization lists epilepsy as a major neurological disease requiring special attention because of its impact on individuals and communities [1]. Epilepsy pathology involves sudden abnormal discharge of neurons in the area of local brain injury, which then spreads to surrounding tissues, resulting in transient brain dysfunction [2]. The International League Against Epilepsy (ILAE) has identified antiseizure medications (ASMs) as the main treatment for epilepsy, especially focal epilepsy, and they remain the preferred first‐line treatment modality [3]. Although existing ASMs are effective for most patients, about 30% of patients develop drug resistance, challenging the effectiveness of drug treatment [4]. Therefore, exploring new therapeutic targets and developing alternative therapies is imperative.

In Mendelian randomization (MR), genetic variation serves as instrumental variables (IVs) to ascertain causal links between modifiable exposures and clinically important outcomes. The fundamental idea relies on Mendelian genetics, using single‐nucleotide polymorphisms (SNPs) as tools to minimize bias from confounding variables and reverse causation inherent in conventional observational research [5, 6]. Furthermore, MR can incorporate publicly available genetic datasets, facilitating data acquisition while effectively minimizing bias from missing individual‐level information [7].

We adopted a systematic analysis framework integrating MR, single‐cell transcriptomic analysis, and molecular docking to identify druggable targets and propose potential treatment strategies for focal epilepsy. The study design comprised two complementary approaches: hypothesis‐driven validation and exploratory discovery. For exploratory discovery, we performed unbiased screening of genome‐wide cis‐expression Quantitative Trait Locus (eQTL) data to identify genes associated with focal epilepsy using MR analysis. To further prioritize potential disease‐related proteins in specific pathways, we conducted co‐localization analysis, summary‐data‐based MR (SMR), the heterogeneity in dependent instruments (HEIDI) test, and linkage disequilibrium score regression. For hypothesis‐driven validation, the expression of prioritized genes was verified in an epilepsy model using western blotting, testing the protein‐level consistency of the genetic predictions. Finally, a comprehensive pharmacological evaluation—including drug–gene interaction analysis and molecular docking—was conducted as an exploratory assessment of the therapeutic potential of candidate targets for focal epilepsy.

## 2 Methods

### 2.1 Data Download

Files containing single‐cell data for accession GSE190452 were sourced from the Gene Expression Omnibus database. Eight samples (four controls and four disease) were analyzed, featuring complete single‐cell expression profiles. Self‐assessed data included expression profile data for eight samples (four controls and four disease). Exposure data: The eQTLGen consortium provided the eQTL data (https://www.eqtlgen.org). The consortium aimed to investigate the genetic architecture of gene expression in blood and its relationship to complex traits. Outcome data: The participants included in this study's outcome‐related genome‐wide association studies (GWAS) were predominantly of European descent. The discovery set (finngen_R12_FE) comprised 9275 cases and 484,703 controls, whereas the validation set (UK Biobank) included 3810 cases and 459, 123 controls.

### 2.2 MR Analysis

SNPs linked to each exposure with a global significance level (*p* < 1e^−5^) [8, 9] were chosen as candidate IVs. SNPs were filtered for linkage disequilibrium, retaining only those with *R* ^2^ < 0.001 within a 10,000 kb clumping window. IVs were considered weak and excluded if their *F*‐value was not above 10. Four statistical approaches were used to evaluate the reliability of causal connections: the inverse variance weighted (IVW) analysis, MR‐Egger regression, weighted median estimator, and weighted model. Wald ratio was applied when only one SNP was implicated in the causal relationship. This method provided a comprehensive estimate of the exposure's impact on focal epilepsy. MR analyses were performed using the “TwoSampleMR” (version 0.6.8).

### 2.3 Multivariate MR (MVMR)

When conducting MVMR analysis, the following points should be noted: Generally, for a SNP, only one exposure factor with a strong correlation (*p* < 1e−5, linkage disequilibrium *r* ^2^ < 0.001) is required. Specific outcome IDs: finngen_R12_G6_CARPTU, finngen_R12_G6_MS, finngen_R12_G6_NERPLEX, finngen_R12_G6_SLEEPAPNO. These four sets of GWAS data for neurological disorders were all obtained from the official FinnGen database.

### 2.4 Sensitivity and SMR Co‐Localization Analysis

We performed leave‐one‐out sensitivity analysis, heterogeneity and pleiotropy assessments. Additionally, co‐localization analysis was conducted using the “coloc” R package (version 5.2.3), and SMR with HEIDI methods were applied to determine whether the SNP effects on phenotype are mediated through gene expression.

### 2.5 Gene Set Enrichment Analysis (GSEA)

Samples were categorized into high‐ and low‐expression groups based on key gene expression levels, and GSEA was used to identify between‐group signaling pathway differences. GSEA was performed using the “clusterProfiler” R package (version 4.10.0). The annotated gene sets for subtype pathway analysis were obtained from the Molecular Signatures Database (MsigDB), version 7.0. The expression of different pathways was assessed, and gene sets with an adjusted *p*‐value of < 0.05 were ranked based on their consistency scores.

### 2.6 Gene Set Variation Analysis (GSVA)

By assessing predefined gene sets, GSVA translates gene‐level variations into pathway‐level insights, revealing a sample's biological function. GSVA was conducted using the “GSVA” R package (version 1.50.0). We utilized the GSVA algorithm to score gene sets obtained from the MsigDB to evaluate potential functional differences across samples.

### 2.7 Transcription Factor Regulatory Network

We used the R package “RcisTarget” to predict transcription factors, which identify enriched DNA‐binding motifs in gene sets. Based on the total motifs in the reference database, the normalized enrichment score was determined for each motif. To estimate motif overrepresentation within a gene set, we first calculated the area under the curve (AUC) for each motif‐gene set pair based on the recovery curves of the gene set‐ranked motifs.

### 2.8 Single‐Cell Transcriptomic Analysis

First, expression profiles were imported using the Seurat package. Data quality was comprehensively considered across multiple samples, and outliers and low‐quality cells with fewer than 200 genes were filtered out. Doublets were further filtered using the DoubletFinder package, ultimately retaining 59,291 high‐quality cells for subsequent analysis.

### 2.9 Molecular Docking

Molecular docking analysis, as an exploratory verification method, is used to evaluate the potential interactions between the protein products of key genes and candidate drugs, providing supportive evidence at the structural biology level for drug repositioning. Nine independent docking simulations were carried out using AutoDock software for molecular docking. The docking result with the lowest binding energy was selected for further analysis. Docking results were imported into PyMOL for visualization.

### 2.10 Building the Animal Model for Epilepsy

Sprague–Dawley male rats (weight range: 240–260 g) were utilized. We constructed the temporal lobe epilepsy (TLE) rat model using the previous method [10]. Rats were sacrificed 1 week later, and hippocampi were rapidly extracted. Only male Sprague–Dawley rats were used in this study. This decision was based on the well‐established effects of sex hormones on neuronal excitability and seizure susceptibility. Estrogen has been reported to exert proconvulsant effects, while progesterone demonstrates anticonvulsant properties [11, 12, 13, 14, 15, 16]. However, the effects of these hormones are not singular or fixed but are influenced by multiple factors, including endocrine status, relative concentrations, and metabolism [11]. To ensure consistency and reliability of the findings by minimizing endocrine‐related variability, we selected male subjects for this initial investigation.

### 2.11 Western Blot

Rat hippocampal samples were lysed in buffer supplemented with protease and phosphatase inhibitors at a 1× concentration. Subsequently, electrophoresis was carried out, and the gel was transferred onto a polyvinylidene fluoride membrane, which was blocked with 5% skimmed milk powder and incubated with specific antibodies. The primary antibodies used were those against: CASP1 (1:1000, Proteintech), FST (1:5000, Proteintech), IL10RA (1:1000, Proteintech), SUCNR1 (1:1000, MyBioSource), and ACTIN (1:1000, Servicebio). The secondary antibodies used were HRP‐conjugated Anti‐Rabbit IgG (H + L) (1:10000, Proteintech) and HRP‐conjugated Anti‐Mouse IgG (H + L) (1:10000, Proteintech).

### 2.12 Statistical Analysis

All statistical analyses, including MR analyses, co‐localization, SMR and HEIDI were conducted in R (version 4.3.0). Western blot data quantification and statistical comparisons were performed using GraphPad Prism 10.1. All data underwent normality testing using Shapiro–Wilk test. For data that followed a normal distribution, parametric tests (e.g., unpaired *t*‐test) were applied. For data that did not meet the assumption of normality, corresponding non‐parametric tests (e.g., Welch *t*‐test) were employed and *p* < 0.05 was considered statistically significant. In Western blot analysis, each group (control group and epilepsy model group) used a total of 3 animals (*N* = 3) as biological repetitions. All experiments are repeated independently at least three times. Use SWE Image Gray Analysis Software (version 3.3.7) to quantify the strength of the protein strip, using β‐actin as the internal reference. The relative expression level of the target protein is calculated according to the ratio of the target protein to β‐actin, and standardized with the control group.

## 3 Results

### 3.1 Discovery MR Analysis

**FIGURE 1:**
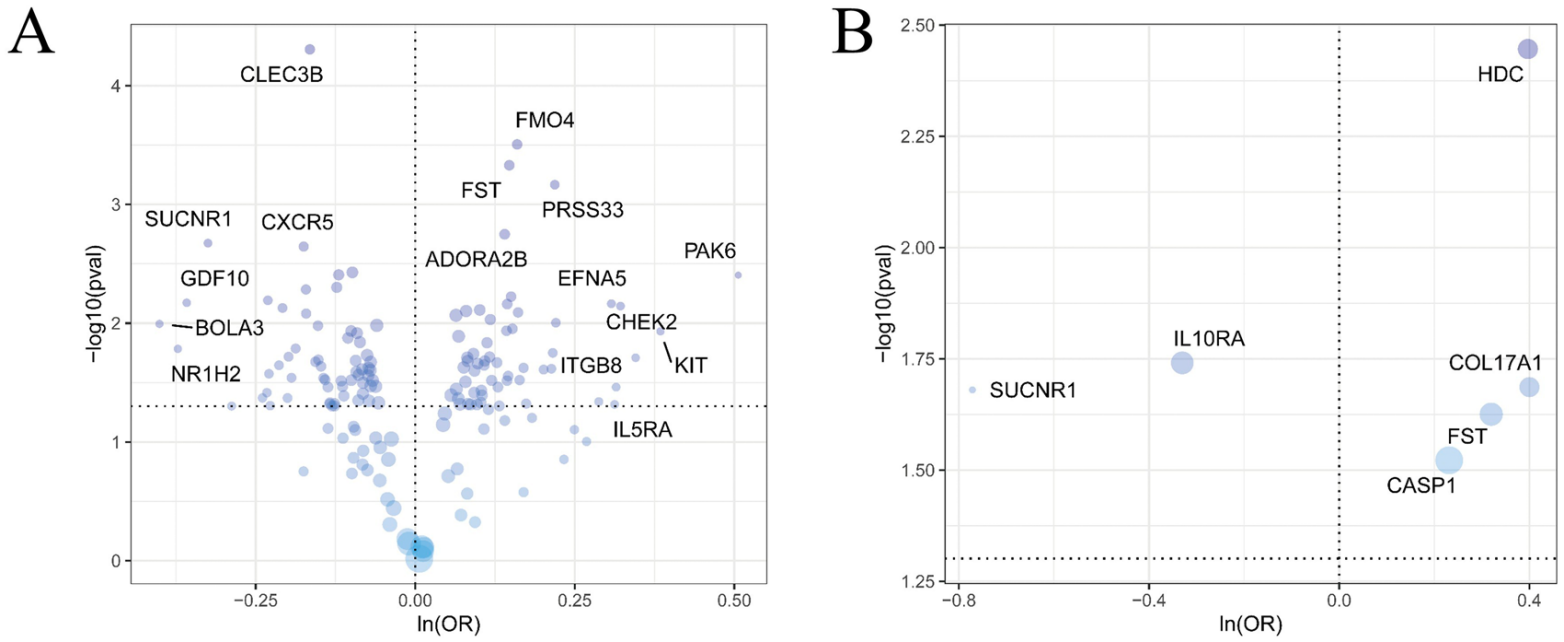
MR of the training set and the validation set. (A) MR was used to obtain the distribution of 157 pairs of causal risk ratios and *p*‐values. (B) MR was used to obtain the distribution of hazard ratios and *p*‐values of six pairs of causal relationships.

MR analysis revealed that 87 gene pairs were significantly associated with eQTL‐positive outcomes (Figure 1A, IVW *p* < 0.05). Among these, 44 genes were associated with reduced focal epilepsy risk (OR < 1), including SUCNR1, CD70, and MADCAM1; the remaining 43, such as PRSS33, EVI2B, and IL5RA, were linked to increased risk (OR > 1). The 87 positive genes with IVW *p* < 0.05 obtained from the finngen_R12_FE were all included in the UK Biobank cohort for repeated MR analysis. Only 6 genes successfully replicated significant causal association signals in the independent validation set, while the remaining 81 genes were excluded from the subsequent validation analysis because no significant causal association (IVW *p* ≥ 0.05) was detected in the validation set.

### 3.2 Validation MR Analysis

To further identify genes that affect focal epilepsy, the outcome id was obtained from the UK Biobank cohort. Further MR analysis was performed to screen out the causal relationship of six pairs of genes (Figure 1B, IVW *p* < 0.05). Results revealed that the presence of SUCNR1 (0.462; 0.240–0.890; *p* = 0.021) and IL10RA (0.719; 0.547–0.945; *p* = 0.018) may be associated with a focal epilepsy risk, while that of CASP1 (1.260; 1.023–1.553; *p* = 0.030), FST (1.377; 1.044–1.816; *p* = 0.024), HDC (1.487; 1.139–1.943; *p* = 0.004), and COL17A1 (1.492; 1.063–2.093; *p* = 0.021) may be associated with a high focal epilepsy risk.

### 3.3 Inverse MR, Sensitivity, and SMR Analysis

In inverse MR, we used finngen_R12_FE as the exposure factor and selected SUCNR1, IL10RA, CASP1, HDC, FST, and COL17A1 as the outcome data. Inverse MR analysis showed that finngen_R12_FE had no causal effect on the eQTL levels of these six genes. Next, we used the SMR tool to perform colocalization analysis on the positive eQTL‐outcome causal relationship pairs. The SMR analysis yielded P_SMR < 0.05 for CASP1, FST, IL10RA, and SUCNR1, while the HEIDI test yielded P_HEIDI > 0.05 for CASP1, COL17A1, FST, IL10RA, and SUCNR1. Therefore, CASP1, FST, IL10RA, and SUCNR1 will be used as key genes for subsequent analysis.

### 3.4 MVMR Analysis and Full Phenotype Group Association Analysis

In a MVMR analysis, CASP1, FST, IL10RA, and SUCNR1 were used as exposure factors, and four neurological diseases were selected as outcome data. The results showed that, under the combined effect, SUCNR1 may have a greater impact on carpal tunnel syndrome than other key genes; SUCNR1, CASP1, and FST may be involved in the disease progression of multiple sclerosis; SUCNR1 may affect the progression of nerve, nerve root, and plexus diseases; and SUCNR1, CASP1, and FST may affect sleep apnea progression. The results showed that CASP1 was associated with a lower risk of dysthymic disorder, vascular dementia, and hydrocephalus. FST was associated with a lower risk of agoraphobia, social phobia, panic disorder, and Parkinson's disease, and a higher risk of alcoholism, adjustment reaction, vascular dementia, somatoform disorder, obsessive‐compulsive disorders, and generalized anxiety disorder. IL10RA was associated with a higher risk of other headache syndromes and specific nonpsychotic mental disorders due to brain damage. SUCNR1 was associated with a lower risk of speech and language disorder, and a higher risk of facial nerve disorders, phobia, hydrocephalus, myoneural disorders, myasthenia gravis, and nerve plexus lesions (Figure S1A–D).

### 3.5 GSEA Analysis

**FIGURE 2:**
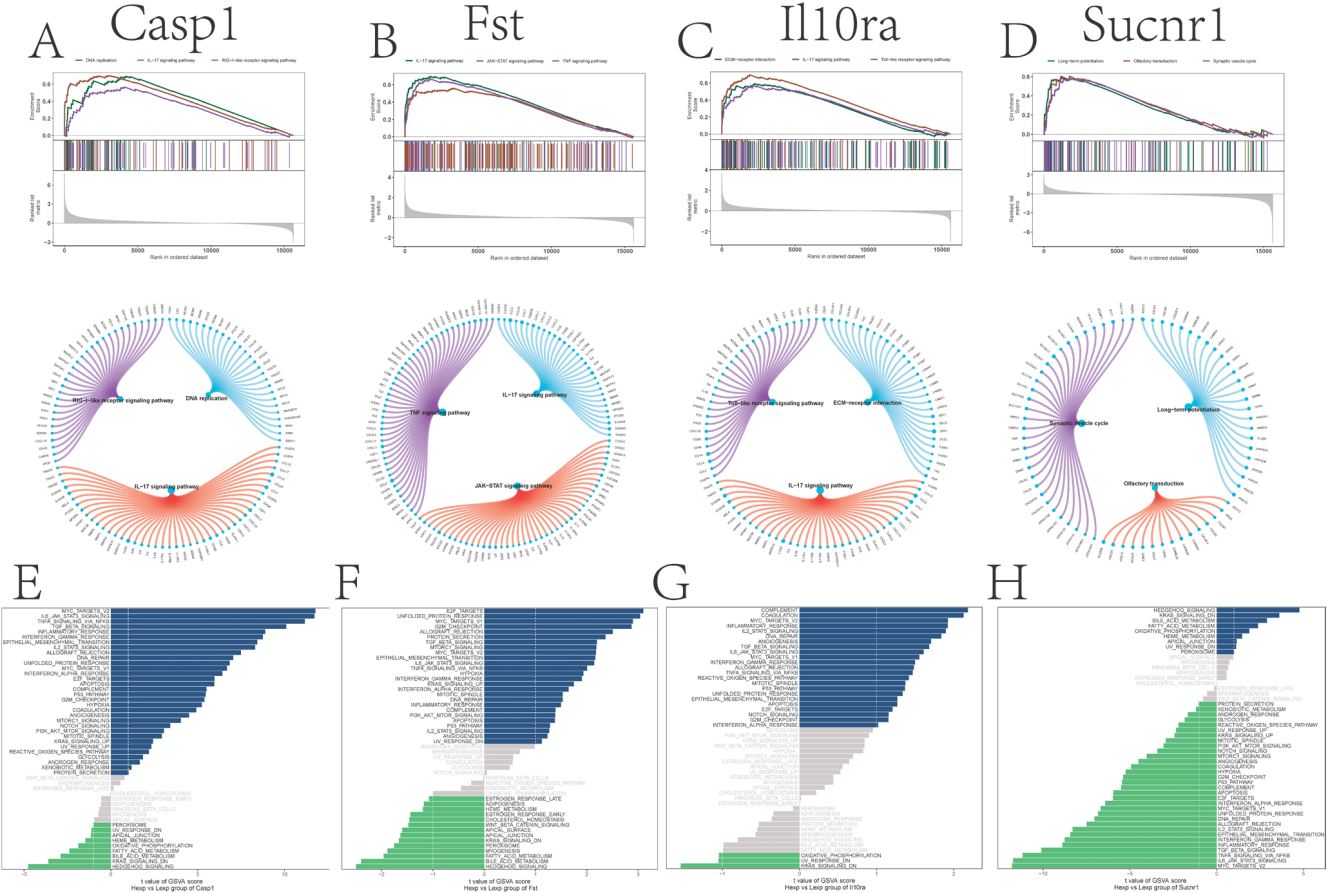
Analysis of key genes GSEA and GSVA. (A–D) GSEA signaling pathways involved in key genes, as well as the regulation of these pathways and the genes involved. (E–H) GSVA analysis of key genes: blue indicates the signaling pathways involved in high gene expression, green indicates the signaling pathways involved in low gene expression, and the background gene set is hallmark.

GSEA results showed that CASP1 was enriched in signaling pathways including the IL‐17 signaling pathway (Figure 2A). FST was enriched in signaling pathways including the TNF signaling pathway (Figure 2B). IL10RA was enriched in signaling pathways including the IL‐17 signaling pathway, ECM‐receptor interaction, and Toll‐like receptor signaling pathway (Figure 2C). SUCNR1 was enriched in signaling pathways such as the synaptic vesicle cycle and long‐term potentiation (Figure 2D).

### 3.6 GSVA Analysis

GSVA analysis showed that CASP1 was enriched in signaling pathways including MYC_TARGETS_V2 and IL6_JAK_STAT3_SIGNALING (Figure 2E). FST was enriched in signaling pathways including E2F_TARGETS and UNFOLDED_PROTEIN_RESPONSE (Figure 2F). IL10RA was enriched in signaling pathways including COMPLEMENT and COAGULATION (Figure 2G). SUCNR1 was enriched in signaling pathways including HEDGEHOG_SIGNALING and KRAS_SIGNALING_DN (Figure 2H).

### 3.7 Transcription Factor Regulatory Network

**FIGURE 3:**
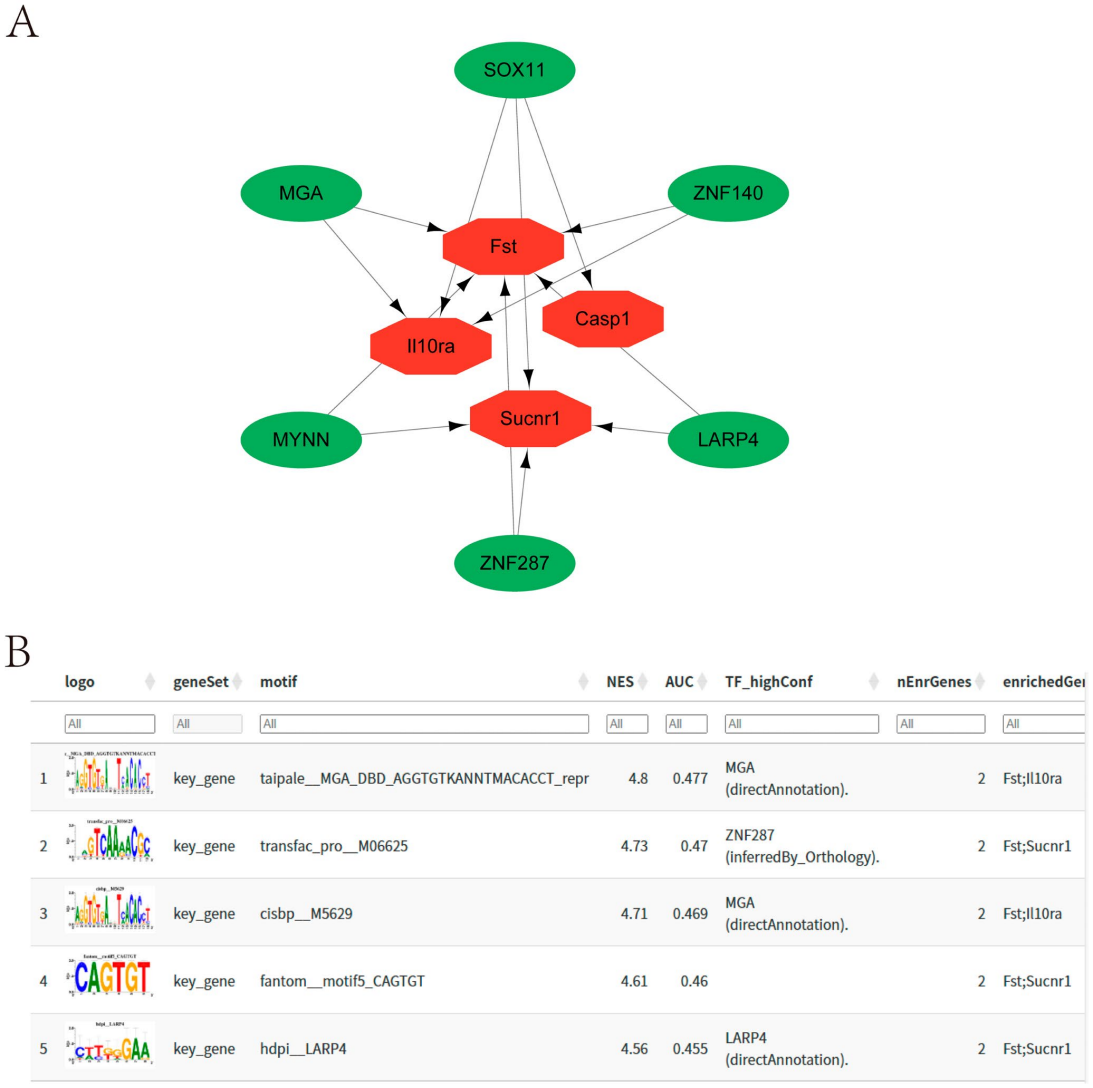
Transcriptional regulation related to key genes. (A) Transcriptional regulatory networks of key genes, with red representing key genes and green representing transcription factors. (B) All the enriched motifs of the key genes and the corresponding transcription factors were presented.

We selected some key genes as the gene set for this analysis, and found that these genes were regulated by a variety of transcription factors. Motif‐TF annotation and selection analysis of important genes revealed that the motif with the highest normalized enrichment score (normalized enrichment score: 4.8) was taipale__MGA_DBD_AGGTGTKANNTMACACCT_repr. Figure 3A,B present the top 10 motifs enriched in key genes and their corresponding transcription factors.

### 3.8 Single‐Cell Transcriptomic Analysis

Figure S2A,B depict the violin plots and scatter plots after filtering. We identified 2000 highly variable genes (Figure S2C). The data were then subjected to normalization, homogenization, Principal Component Analysis (PCA), and harmony analysis (Figure S2D–F). After dimensionality reduction using the UMAP algorithm, all cells were divided into 11 subpopulations (Figure S3A). All the cells were divided into 11 subgroups, and they were labeled as seven cell types based on the known markers (see Figure S3A,B). Compared to the control group, the disease group exhibited a decrease in the proportions of oligodendrocytes (from 29.6% to 21.8%), excitatory neurons (from 30.5% to 25.4%), and inhibitory neurons (from 15.1% to 10.8%), while the proportions of astrocytes (from 10.7% to 16.9%), microglial cells (from 7.7% to 14.4%), oligodendrocyte precursors cells (OPCs) (from 5.5% to 9.5%), and endothelial cells (from 0.9% to 1.2%) were increased. The bubble graphs of these markers and the histograms of the proportions of each group of cells are shown in Figure S3C,D.

### 3.9 Key Gene Expression and Immunometabolism Pathway Activity

**FIGURE 4:**
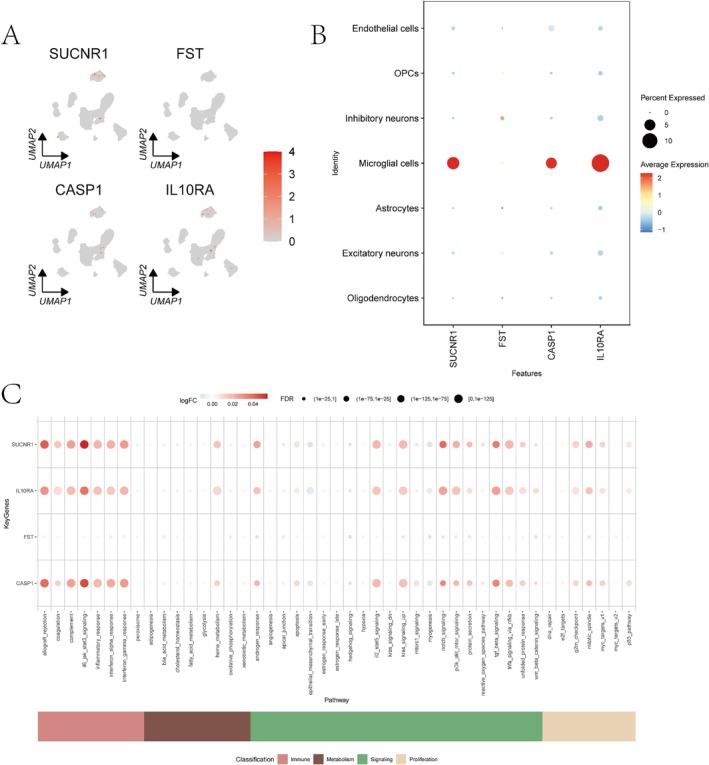
Differences in the expression of key genes in single cells and the activity of immune metabolic pathways. (A) Scatter plot of the expression profile of key genes in a single cell. (B) Bubble chart of the expression profile of key genes in single cells, with blue indicating high expression and red indicating high expression. (C) Differences in the activity of key genes and immune metabolism, with blue indicating low expression and red indicating high expression.

We observed that SUCNR1, CASP1, and IL10RA were all highly expressed in microglial cells (Figure 4A,B). Next, we used AUCell to quantify and score genes in immune‐metabolism pathways in single cells and used bubble plots to display differential expression of key genes in immune‐metabolism pathways. The results showed that SUCNR1, CASP1, and IL10RA were highly active in pathways including allograft rejection, IL6 JAK STAT3 signaling, and tgf beta signaling; while FST was highly active in pathways including hedgehog signaling and myogenesis (Figure 4C).

### 3.10 Drug Prediction and Molecular Docking

**FIGURE 5:**
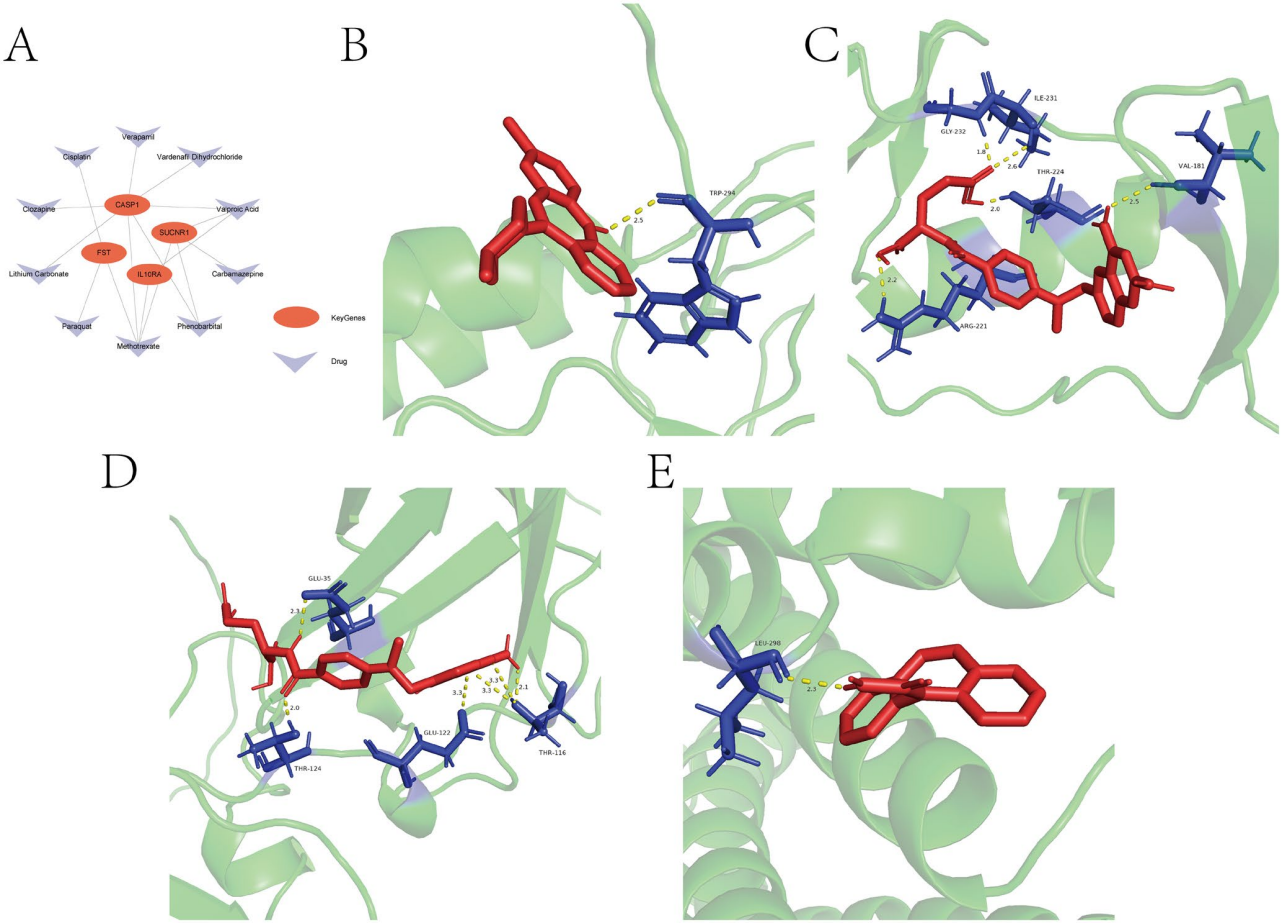
Drug prediction and molecular docking. (A) Interaction network between key genes and drugs predicted based on the CTD database. (B–E) Interaction relationship between key genes and molecules. Each node represents a molecule, and the lines connecting nodes indicate the docking or interaction between molecules.

Using Comparative Toxicogenomics Database (CTD), we found seven drugs interacting with CASP1, three with FST, two with IL10RA, and three with SUCNR1 (Figure 5A). In molecular docking, the proteins and compounds selected for key genes were CASP1: P29466‐Clozapine, FST: P19883‐Methotrexate, IL10RA: Q13651‐Methotrexate, and SUCNR1: Q9BXA5‐Carbamazepine. The molecular docking results showed a binding energy of −6.7 kcal/mol for CASP1: P29466‐Clozapine (Figure 5B); −6.5 kcal/mol for FST: P19883‐Methotrexate (Figure 5C); −6.9 kcal/mol for IL10RA: Q13651‐Methotrexate (Figure 5D); and −6.5 kcal/mol for SUCNR1: Q9BXA5‐Carbamazepine (Figure 5E).

### 3.11 Western Blot

**FIGURE 6:**
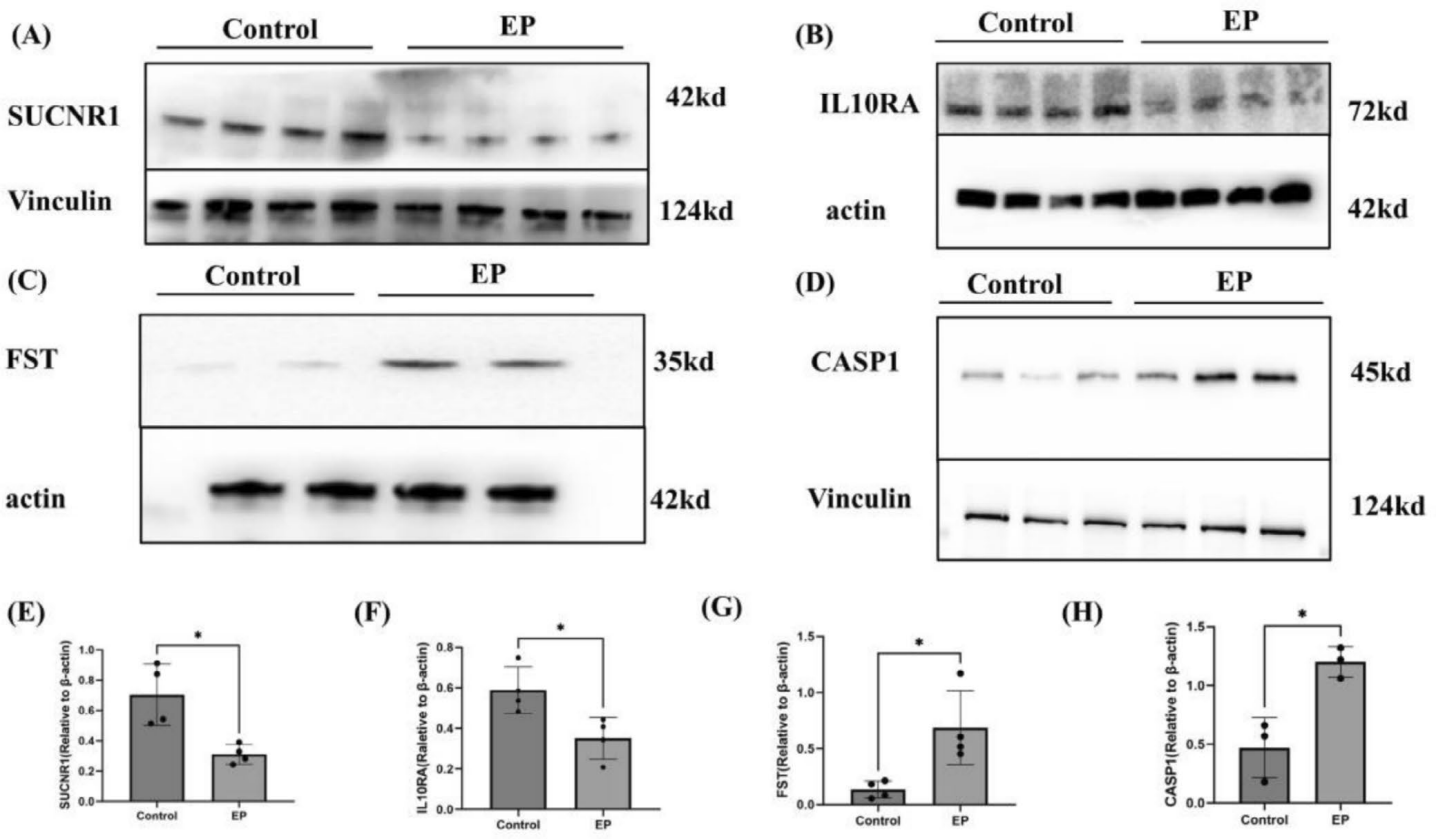
Protein expression of SUCNR1, IL10RA, FST, and CASP1 in epilepsy models. (A–H) Representative western blot images and quantifications of SUCNR1, IL10RA, FST and CASP1 protein expressions in hippocampal tissue, *p < 0.05. EP, epileptic seizure group.

Western blotting revealed the expression of the above four proteins in the hippocampus of epileptic rats. Figure 6 depicts the decreased expressions of SUCNR1 and IL10RA in epilepsy, while there is increased expression of FST and CASP1.

## 4 Discussion

Focal epilepsy accounts for about 60%–70% of all epilepsy cases and is the main epilepsy type. Previous genetic studies have extensively explored voltage‐gated sodium channel genes, including Sodium Voltage‐Gated Channel Alpha Subunit 1 (*SCN1A*) and *SCN2A*, and potassium channel genes, such as Potassium Voltage‐Gated Channel Subfamily Q Member 2 (*KCNQ2*), with mutations in these genes affecting neuronal action potential generation and transmission [17, 18, 19]. While recent MR studies have investigated causal relationships between various risk factors and epilepsy, most have focused on broad exposures rather than systematically screening druggable genes at the transcriptomic level [20, 21]. In the present study, we employed an integrative multi‐omics framework that extends beyond these prior approaches by combining MR with co‐localization analysis, single‐cell transcriptomic analysis, and molecular docking to systematically prioritize druggable genes for focal epilepsy. Apart from the above‐mentioned known pathogenic genes, we further screened and identified a number of potential pathogenic genes, including *CASP1*, *FST*, *IL10RA*, and *SUCNR1*, providing important clues for exploring new treatment targets for focal epilepsy.

ASMs are the mainstay of focal epilepsy treatment. However, drug selection and treatment plan need to be individually adjusted based on patient situation. In addition to drug treatment, surgery is a key option for focal epilepsy treatment, especially for patients with drug‐intractable epilepsy. Recent advancements in genomics have opened up new methodologies for epilepsy research and treatment. Reportedly, focal epilepsy is closely related to a variety of genetic mutations and polymorphisms. Notably, mutations of genes such as SCN1A, SCN2A, and KCNQ2 play an important role in the pathogenesis of epilepsy. For instance, Dravet syndrome, which is a serious hereditary epilepsy, is usually caused by functional deletion mutations of the SCN1A gene [22, 23]. Further, SCN2A gene mutations are associated with a variety of epilepsy diseases, especially in children. These mutations may reduce sensitivity to traditional sodium channel blockers [24]. Epilepsy's genetic research has widely explored voltage‐gated sodium channel genes, including SCN1A and SCN2A, and potassium channel genes, such as KCNQ2. Mutations of these genes affect the generation and transmission of neuronal action potentials, and may lead to various seizure types [17, 25]. For example, KCNQ2 gene variation is associated with growth retardation and a variety of epilepsy phenotypes. Researchers have used machine learning models to classify these mutations to support clinical decision‐making and prognostic evaluation [26]. Apart from the above‐mentioned known pathogenic genes, we further screened and identified a number of potential pathogenic genes, including CASP1, FST, IL10RA, and SUCNR1, providing important clues for exploring new treatment targets for focal epilepsy.

In the pathophysiology of epilepsy, inflammation and programmed cell death (such as death) play a key role. Reportedly, the activation of inflammatory bodies (especially NLRP3 and NLRP1) is closely related to the occurrence and progression of epilepsy. Inflammatory bodies trigger the release of inflammatory mediators including IL‐1β and IL‐18 by activating caspase‐1 to initiate an inflammatory response and burning death [27]. In patients with TLE, both NLRP3 and NLRP1 inflammatory inflammasomes are significantly upregulated, which may lead to an increase in the expression of caspase‐1 and IL‐1β in the hippocampal sclerosis [28]. Caspase‐1 is crucial in the development of epilepsy. Inhibiting caspase‐1 can effectively reduce neuronal death and inhibit inflammatory reactions. The caspase‐1 inhibitor VX‐765 has shown good safety in epilepsy clinical trials and the potential to treat cerebral ischemia in animal studies [29]. Reportedly, the small molecule caspase‐1 inhibitor CZL80 reduces seizures in drug‐resistant TLE models and reduces neuroexcitability in patient brain slices [30].

As a well‐known regulatory protein, FST promotes cell proliferation by interacting with the HIPPO/YAP signaling pathway [31]. Research on myocardial cells shows that FST may play a similar role in promoting the proliferation and repair of nerve cells. Neuronal hypersynchronous discharge and abnormal discharge are key pathological features of epilepsy, leading to neuronal damage and cell death [32]. FST may help reduce neuronal damage associated with epilepsy by regulating cell proliferation and survival signaling pathways. However, most of the current research focuses on FST's role in heart tissue. Furthermore, myocardial cell research shows that FST can significantly improve heart function and reduce scar tissue formation after myocardial infarction [31]. The repair and functional benefits of FST show that it may have broad application in epilepsy treatment. It can promote neuronal regeneration, improve nerve function, and reduce the frequency and severity of seizures. Although further research is needed to determine its direct therapeutic effect on epilepsy, FST's role in other physiological processes shows that it has great therapeutic potential. Future studies clarifying its mechanism of action in the nervous system and evaluating its clinical application in epilepsy treatment are warranted, which may provide a theoretical basis for the development of innovative therapies.

IL10RA is associated with autoimmune and inflammatory diseases, and it may also play a role in neurological diseases. IL10RA encodes a sub‐receptor of the IL‐10 receptor, which is a cytokine receptor that mediates anti‐inflammatory signal transduction. Mutation and polymorphism of IL10RA destroy the function of the receptor and lead to changes in the immune response, which may lead to disease development. Reportedly, IL10RA polymorphism was associated with whiteness, an inflammatory disease with neurological manifestations. This study identified the mononucleotide polymorphism (SNP) rs4936415 located in the IL10RA enhancement subregion, which can regulate IL10RA expression; the protective G allele enhances IL10RA expression, and the risk C allele and NF‐κB1 bind to promote the increase of enhancer activity and IL10RA expression. These findings indicate that IL10RA expression disorder may lead to the inflammatory process and may affect the prognosis of the nervous system [33]. Moreover, IL10RA's role in immune regulation is reflected in its participation in other diseases with neurological symptoms. For example, increased IL10RA expression is associated with the resistance to ALK inhibitors of large cell lymphoma (a malignant tumor that may involve the nervous system). Increased IL10RA expression can reshape the STAT3 signaling pathway and bypass key phosphorylation events, which may affect the prognosis of the nervous system of affected patients [34].

SUCNR1 is receiving increasing attention in epilepsy research. As a G protein‐coupled receptor, SUCNR1 mainly acts by sensing succinic acid (an intermediate product of the tricarboxylic acid cycle). SUCNR1 is crucial in a variety of pathological conditions, including hypertension, ischemia–reperfusion injury, inflammation, and immune response [35]. Moreover, the positioning and transport mechanism of SUCNR1 in the nervous system are being studied in depth, and its positional changes on the endoplasmic reticulum and cell membrane are closely related to cell stress response [36]. In neurons, the role of SUCNR1 involves its expression and functional translocation. Under hypoxic conditions, SUCNR1 is transferred from the endoplasmic reticulum to the cell membrane, which is related to the regulation of neuronal excitability [36]. SUCNR1 activation is also related to the regulation of inflammatory reactions, which may indirectly affect neuron excitability and seizure susceptibility [37]. In summary, these results show that SUCNR1 may play a key role in the pathophysiology of epilepsy. Moreover, SUCNR1 is involved in the physiological processes of a variety of non‐neuron cell types. For example, SUCNR1 activation in skeletal muscle cells can enhance oxidative phosphorylation and improve muscle explosiveness [38]. This may affect neuronal function by regulating intracellular energy metabolism, which may cause seizures. SUCNR1 activation in intestinal epithelial cells is related to the epithelial‐mesenchymal transformation, which is related to fistula formation in Crohn's disease [39]. SUCNR1 may affect epilepsy through a variety of mechanisms, including the direct regulation of neuronal excitability and indirect effects mediated by inflammatory response and energy metabolism. Although the exact mechanism remains unclear, existing evidence supports further study of SUCNR1 as a new therapeutic target; future studies should explore its role in different types of epilepsy and its potential as a drug target.

Notwithstanding the value of the findings of this study, there are a number of limitations that should be noted. (1) The genetics analysis was based on a limited population, that is, individuals of European descent. In future research, diverse populations should be studied to obtain more comprehensive results. (2) We employed an exploratory screening strategy to identify potential therapeutic targets for epilepsy. Although we enhanced the reliability of the results through various measures—such as strict exposure screening criteria, quality control of instrumental variables, and colocalization analysis—no formal multiple testing correction was implemented during the initial MR screening stage. Consequently, there is still the possibility of false positives in the association results. Conversely, strict correction methods may increase the risk of false negatives, reducing the number of potentially important targets. Therefore, the absence of formal multiple testing correction remains a statistical limitation of the present analysis. (3) The drug–gene interaction analysis and molecular docking results provided supportive mechanistic insights, highlighting the molecular relevance and therapeutic potential of the identified targets; however, it is important to emphasize that these findings represent hypothesis‐generating in silico evidence rather than definitive proof of efficacy. The observed moderate binding affinities suggest plausible interactions but do not confirm functional activity, which warrants further experimental validation through preclinical and clinical studies, particularly for drugs not routinely used in epilepsy treatment. (4) In constructing the animal model to verify the identified target genes, we used only male rats; therefore, it is possible that there are sex differences not uncovered by our analysis. (5) Western blot validation experiments confirmed the consistency of the expression patterns between genetic predictions and animal models, providing supportive evidence at the protein level; however, these experiments did not establish direct functional causality. Further studies through genetic manipulation or drug intervention are still needed to confirm the causal role of these targets in the occurrence of epilepsy. Considering these limitations, the results of this study should be regarded as hypothesis‐generating discoveries rather than proof of treatment efficacy. Further validation is needed through the use of larger‐scale independent cohorts and functional experiments.

## 5 Conclusions

CASP1, FST, IL10RA, and SUCNR1 were identified as potential therapeutic targets for focal epilepsy through integrative MR and single‐cell sequencing analyses. These findings provide novel insights into the genetic and molecular mechanisms of focal epilepsy and highlight promising avenues for drug development. Further research is needed to validate these targets across diverse populations and explore their translational potential in clinical settings.

## Author Contributions

**Huaiyu Sun:** software development, data curation, and original draft writing. **Xuewei Li:** conceptualization, investigation, and drafting of the original manuscript. **Weixuan Zhao:** investigation and data curation. **Hongmei Meng:** formal analysis, investigation, and writing – review and editing. **Wuqiong Zhang:** supervision, formal analysis, writing – review and editing, and funding acquisition. All authors reviewed and approved the final manuscript.

## Funding

This study received financial support from the Department of Science and Technology of Jilin Province (20240304165SF) and the Education Department of Jilin Province (2025KC102).

## Ethics Statement

All animal experiments in this research were carried out in full accordance with international Animal Care Guidelines and were sanctioned by the Animal Ethics Committee of the First Hospital of Jilin University (JDYY20240457).

## Consent

The authors have nothing to report.

## Conflicts of Interest

The authors declare no conflicts of interest.

## Supporting information


**Figure S1:** MVMR and full phenome association.
**Figure S2:** Single‐cell pretreatment.
**Figure S3:** Cell annotation.

## Data Availability

All data within the study are presented in the paper and/or the Supporting Information. The original data and additional data related to this study are available from the corresponding author upon reasonable request.
