# Supplementary material for: Integrative Drug‐Target Causal Analysis, Single‐Cell Sequencing and In‐Vivo Validation for Dissecting Molecular Mechanisms Underlying Focal Epilepsy
Source: CNS Neurosci Ther. 2026 Jun 15;32(6):e70985. doi: 10.1002/cns.70985 (PMC13267665; doi:10.1002/cns.70985)
Supplement: Supplementary file 1 — Figure S1: MVMR and full phenome association. Figure S2: Single‐cell pretreatment. Figure S3: Cell annotation. [file CNS-32-e70985-s001.docx]

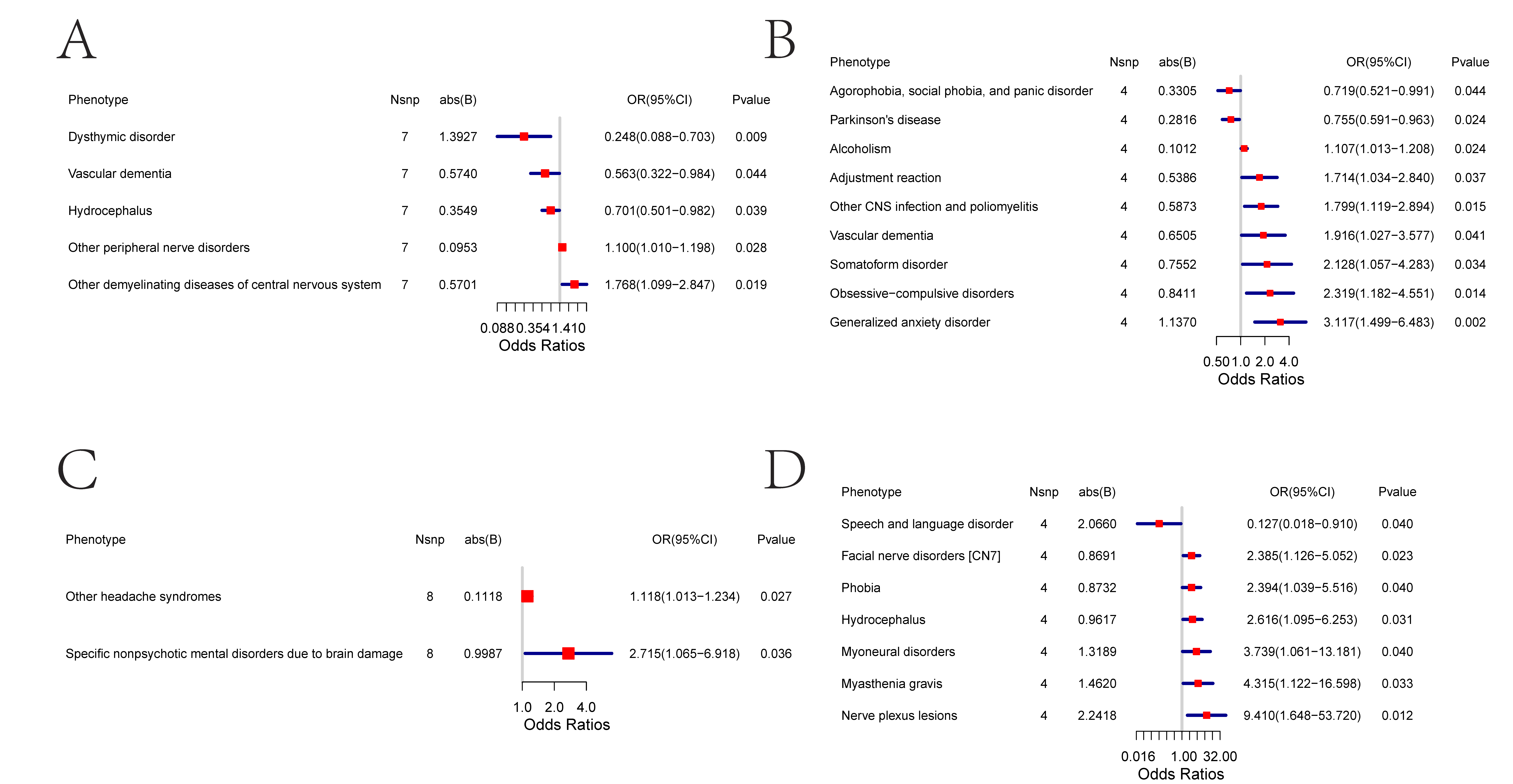


Supplementary Figure 1. MVMR and full phenome association

(A-D) MVMR was used to obtain the distribution of hazard ratios and p-values for each causal relationship.


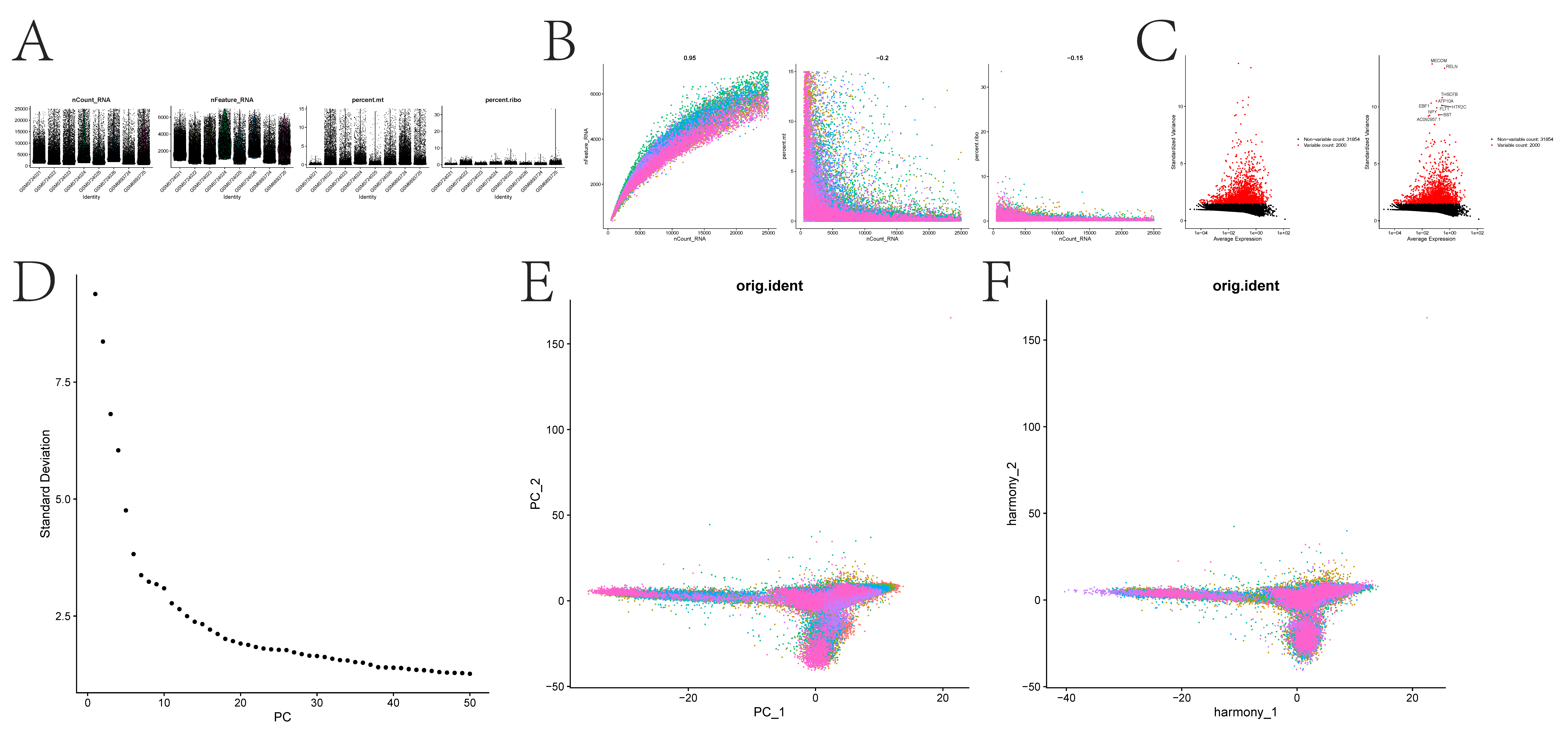


Supplementary Figure 2: Single-cell pretreatment

(A) Single-cell quality control, showing the number of cells, genes and sequencing depth for each sample.

(B) The left figure shows the relationship between cell sequencing depth and mitochondrial content, and the middle figure shows the relationship between plasmid content and nCount_RNA. The right figure shows the relationship between sequencing depth and the number of genes. The scatter plot shows the correlation between mitochondrial content (Y-axis) and nCount_RNA (X-axis). Each point represents a cell, indicating the distribution of RNA counts relative to mitochondrial gene expression levels.

(C) We identified genes that showed significant differences among cells and plotted characteristic variance plots.

(D) Variance sorting plot of each PC.

(E-F) Display of PCA and distribution of PC, with dots representing cells and colors representing samples.





Supplementary Figure 3. Cell Annotation

(A) Based on the important components available in PCA, we divided the cells into 11 clusters through the UMAP algorithm.

(B) Cell annotation situation of 11 clusters, 11 clusters were annotated into 7 cell types Namely Oligodendrocytes, Excitatory neurons, Astrocytes, Microglial cells, Inhibitory neurons, and OPCs, Endothelial cells.

(C) Bubble plots of doplots for seven types of cells and cell markers.

(D) The differences in the proportion of seven types of cells in the two groups of samples.
